# The Arabidopsis Iron-Sulfur (Fe-S) Cluster Gene *MFDX1* Plays a Role in Host and Nonhost Disease Resistance by Accumulation of Defense-Related Metabolites

**DOI:** 10.3390/ijms22137147

**Published:** 2021-07-01

**Authors:** Jose Pedro Fonseca, Sunhee Oh, Clarissa Boschiero, Bonnie Watson, David Huhman, Kirankumar S. Mysore

**Affiliations:** 1 Noble Research Institute, Ardmore, OK 73401, USA; zepedrof@gmail.com (J.P.F.); sunnypage@hotmail.com (S.O.); clarissaboschi@yahoo.com (C.B.); bonniewatson2016@gmail.com (B.W.); david.huhman@gmail.com (D.H.); 2 Institute for Agricultural Biosciences, Oklahoma State University, Ardmore, OK 73401, USA; 3 Department of Biochemistry and Molecular Biology, Oklahoma State University, Stillwater, OK 74078, USA

**Keywords:** mitochondria, ferredoxin, MFDX1, NFS1, biotic stress, abiotic stress

## Abstract

Until recently, genes from the iron-sulfur (Fe-S) cluster pathway were not known to have a role in plant disease resistance. The *Nitrogen Fixation S (NIFS)-like 1* (*NFS1*) and *Mitochondrial Ferredoxin-1* (*MFDX1*) genes are part of a set of 27 Fe-S cluster genes induced after infection with host and nonhost pathogens in Arabidopsis. A role for *AtNFS1* in plant immunity was recently demonstrated. In this work, we showed that *MFDX1* is also involved in plant defense. More specifically, Arabidopsis *mfdx1* mutants were compromised for nonhost resistance against *Pseudomonas syringae* pv. *tabaci*, and showed increased susceptibility to the host pathogen *P. syringae* pv. *tomato* DC3000. Arabidopsis *AtMFDX1* overexpression lines were less susceptible to *P. syringae* pv. *tomato* DC3000. Metabolic profiling revealed a reduction of several defense-related primary and secondary metabolites, such as asparagine and glucosinolates in the Arabidopsis *mfdx1-1* mutant when compared to Col-0. A reduction of 5-oxoproline and ornithine metabolites that are involved in proline synthesis in mitochondria and affect abiotic stresses was also observed in the *mfdx1-1* mutant. In contrast, an accumulation of defense-related metabolites such as glucosinolates was observed in the Arabidopsis *NFS1* overexpressor when compared to wild-type Col-0. Additionally, *mfdx1-1* plants displayed shorter primary root length and reduced number of lateral roots compared to the Col-0. Taken together, these results provide additional evidence for a new role of Fe-S cluster pathway in plant defense responses.

## 1. Introduction

Iron-sulfur (Fe-S) clusters are cofactors associated with proteins that can mediate electron transfer, enzymatic catalysis, and other plant physiological processes such as development, amino acid metabolism, photosynthesis, and respiration [1,2,3,4,5]. They are formed by iron atoms and inorganic sulfide. In eukaryotes, Fe-S clusters are formed and utilized in the mitochondria and then distributed throughout the cell [2]. The assembly of Fe–S clusters and insertion on polypeptide chains require several dedicated gene-coding proteins inside the living cell and so far around forty-six genes have been identified in Arabidopsis [6]. In plants, there are mainly three pathways involved in the Fe-S cluster assembly process: the sulfur mobilization (SUF) pathway in plastids, iron-sulfur cluster (ISC) pathway in mitochondria, and cytosolic Fe-S protein assembly (CIA) in the cytosol [7]. The similarities found so far between prokaryotic and eukaryotic Fe-S assembly pathways suggest that these pathways were inherited in plants by endosymbionts. The ISC assembly pathway is very similar to the nitrogen-fixation-specific (NIF) pathway in nitrogen fixing bacteria. Similarly, the SUF pathway bears resemblance with bacterial sulfur mobilization pathway [7]. The current working model suggests that the assembly process from all three pathways can be summarized in three main steps: First, generation of sulfur in the form of sulfide (S) from cysteine residues by dedicated enzymes such as cysteine desulfurases (e.g., At5g65720, *AtNFS1*); A second step, where S and Fe are combined on a scaffold protein; A third step, where the Fe-S cluster is transferred to a target protein [6]. Additionally, Fe-S clusters can have a regulatory role in biological processes. For example, in bacteria Fe-S clusters can act as oxidative stress sensors [8].

Several Fe-S cluster proteins contribute to electron transfer in the respiratory electron transport chain [8,9,10]. The function of several genes involved in mitochondrial and cytosolic Fe-S protein assembly remains largely unknown. Mitochondrial ferredoxins (MFDX1 and MFDX2), as well as a Ferredoxin oxidoreductase (MFDR) are part of the electron transfer chain in the mitochondria that provides reducing power from electrons for the cell [11]. Similarly, cytosolic proteins of the Fe-S cluster machinery like the Cytosolic iron-sulfur protein assembly 1 (CIA1) depends on the assembly of the clusters in the mitochondria [2] in order to provide Fe-S clusters to proteins in the cytosol and the nucleus. The Monothiol glutaredoxin (GRX17) protein associates with several members of the cytosolic Fe-S cluster machinery, including CIA1, and has a role in DNA damage and resistance to the fungus *Botrytis cinerea*, suggesting a role in plant defense [12]. Recently, we showed the role of two Fe-S cluster proteins Nitrogen fixation S (NIFS)-like 1 (NFS1) and its interactor Frataxin (FH) in plant immunity [13]. Briefly, Arabidopsis *atnfs1* and *atfh* mutants were more susceptible against host and nonhost pathogens, while *AtNFS1* and *AtFH* overexpression lines displayed decreased susceptibility to infection by the host pathogen *Pseudomonas syringae* pv. *tomato* DC3000 [13]. The *AtNFS1* overexpression line also displayed constitutive upregulation of several defense-related genes.

Ferredoxins are small Fe-S cluster proteins that are major distributors of redox potential in plants. The role of chloroplastic ferredoxins (FD) in plants has been well studied. They function as major electron sinks and donors in the chloroplastic electron transport chain and therefore affect a number of metabolic reactions such as photosynthesis, sulfur, and nitrogen assimilation, mainly in redox signaling [14,15,16]. The plastidial ferredoxin mutant *atfd2* was shown to be more susceptible to the host pathogen *P. syringae* pv. *tomato* DC3000 and displayed increased levels of jasmonic acid [17]. In contrast, the role of mitochondrial ferredoxins (MFDX1, MFDX2, and MFDR) remains largely unknown. It was previously shown that mitochondria-localized recombinant AtMFDX1 and AtMFDR proteins can transfer electrons from NAD(P)H to cytochrome C in vitro, indicating a role for these genes in the redox metabolic pathway in plants [11].

In plants, the biological role of Fe-S proteins in biotic and abiotic stress is emerging [12,13,18]. Plants have evolved different and integrated mechanisms to perceive pathogens and defend against them. Basal resistance is the first layer of defense responses where plants recognize pathogen-associated molecular patterns (PAMPs) to trigger a defense response called PAMP triggered immunity. A second layer of defense called host resistance or gene-for-gene resistance is mediated by a network of plant disease resistance (R) proteins that are responsible for detection of pathogen (host pathogen) secreted effectors to trigger a stronger defense response known as effector triggered immunity. Another type of resistance known as nonhost resistance (NHR) is shown by a given plant species against microbes that are not pathogenic to them (nonhost pathogens), but are pathogenic in other plant species [19,20]. Nonhost resistance (NHR) can be used to generate durable disease resistance in plants [20,21,22]. Several genes involved in NHR have been used to generate resistance in economically important crops against pathogens [23]. A useful and time-saving tool for the characterization of genes involved in NHR is the downregulation of the expression of a target gene in *N. benthamiana* by using *Tobacco rattle virus* (TRV)-based virus-induced gene silencing (VIGS) [24]. This approach has been successfully implemented previously in the study of *NbNFS1* [13].

In this work, we demonstrated a previously unknown role for the Fe-S cluster *mitochondrial ferredoxin-1* (*MFDX1*) gene in NHR and basal resistance. When compared to the wild-type (WT) plant, Arabidopsis *mfdx1* mutants were more susceptible to host and nonhost pathogens. In contrast, Arabidopsis *MFDX1* overexpression lines were partially resistant against a host pathogen. In addition, metabolic profiling of an Arabidopsis *AtNFS1* overexpression line and the *mfdx1* mutant upon host pathogen infection revealed differential accumulation of several defense-related primary and secondary metabolites.

## 2. Results

### 2.1. Several Fe-S Cluster Genes Are Upregulated in Arabidopsis Upon Host and Nonhost Pathogen Infection

**Figure 1:**
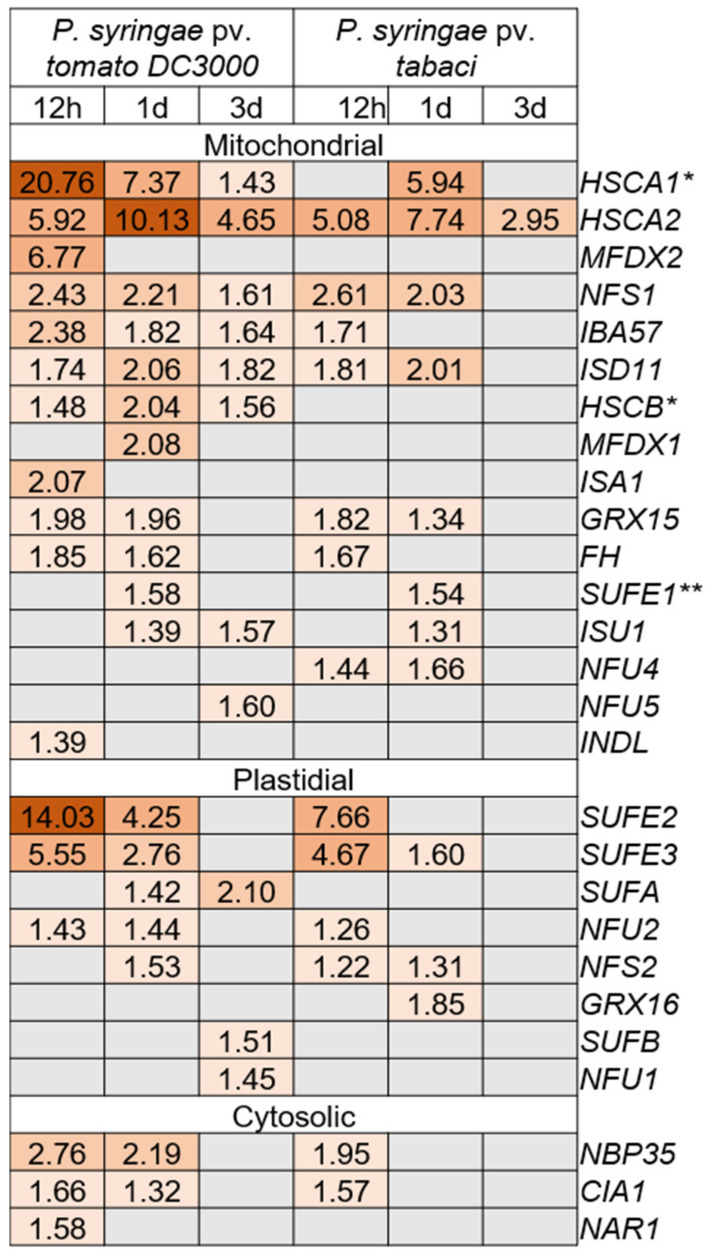
RNA-seq analysis showing upregulation of 27 Fe-S cluster pathway genes in WT plants upon host and nonhost pathogen infection: Heatmap showing differentially expressed genes with their respective fold-change from 27 upregulated Fe-S cluster genes upon host (*P. syringae* pv. *tomato* DC3000) and nonhost (*P. syringae* pv. *tabaci*) pathogen infection in the WT. Differentially expressed genes at 12 h, 1 day, and 3 days were required to have a False-Discovery Rate (FDR) <0.05 compared to the 0 h time point control. The predicted localization of proteins encoded by these genes are shown as plastids or mitochondria or cytosol. Upregulated genes are color coded from light to dark red according to increasing fold-change values. * Gene that encodes a protein that also localizes in the cytosol. ** Gene that encodes a protein that also localizes in the plastid.

Previously, we have shown that several proteins from the Fe-S cluster pathway, including the NFS1 and its interactor FH, are involved not only in nonhost disease resistance but also in the basal resistance of Arabidopsis [13]. To investigate how many Fe-S cluster genes are induced upon *P. syringae* pv. *tomato* DC3000 infection, we performed an RNA-seq experiment of Arabidopsis (Col-0 ecotype; WT) after inoculation with host or nonhost pathogen. Briefly, 4–5 week-old Col-0 plants were flood inoculated [25] with a host pathogen, *P. syringae* pv. *tomato* DC3000 (CFU = 8 × 10^4^), and a nonhost pathogen, *P. syringae* pv. *tabaci* (CFU = 1.6 × 10^6^). The aerial part of seedlings was collected from three plants (*n* = 3) at different time points (0 h post inoculation (hpi), 12 hpi, 1 day post inoculation (dpi), and 3 dpi) after pathogen inoculation. Total RNA was isolated from these samples. Individual RNA-seq libraries were prepared and uniquely indexed for Illumina sequencing. Later, we mined the RNA-seq data for differentially expressed known Fe-S cluster genes at 12 h, 1 dpi, and 3 dpi compared to control (0 hpi) samples using a stringent false discovery rate (FDR) <0.05. From a total of around 40 known Fe-S cluster genes in Arabidopsis, a total of 27 were induced for at least one time point, either by a host or a nonhost pathogen, compared to 0 hpi (Figure 1). Proteins encoded by the majority of these genes including *MFDX1* and *NFS1* are predicted and/or shown to localize in mitochondria. Among the proteins encoded by rest of the differentially expressed Fe-S cluster genes, eight are predicted to localize in chloroplast and three in cytosol (Figure 1).

### 2.2. The Fe-S Cluster Gene AtMFDX1 Contribute to Host and Nonhost Resistance

Previously, we have shown that the Fe-S cluster genes *NFS1* and *FH*, when silenced in *Nicotiana benthamiana*, compromised nonhost resistance to *P. syringae* pv. *tomato* T1 [13]. We also showed that an additional 11 Fe-S cluster genes, including a *MFDX1* (Figure S1), when silenced in *N. benthamiana* by VIGS, displayed increased susceptibility to a nonhost pathogen.

**Figure 2:**
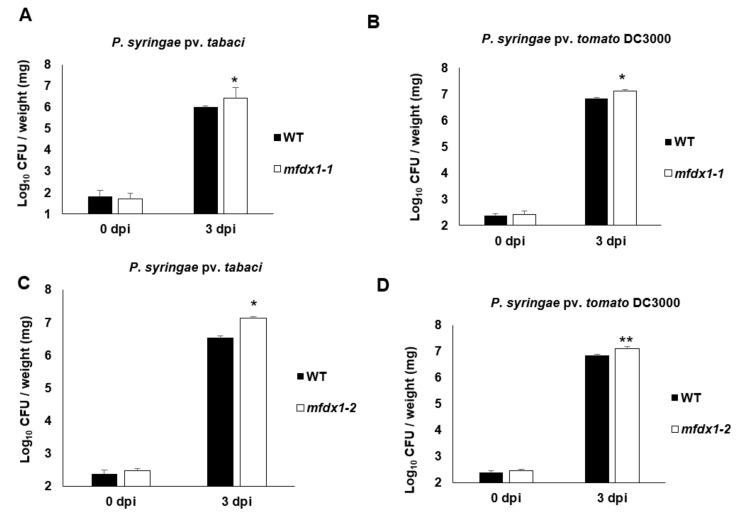
The Arabidopsis *mfdx1* mutants are more susceptible to host and nonhost pathogen infection: (**A**,**B**) Quantification of host and nonhost pathogen multiplication in the *mfdx1-1* mutant. Three-week-old WT (Col-0) and *mfdx1-1* mutant plants were flood inoculated with nonhost pathogen *P. syringae* pv. *tabaci* at 1.6 × 10^6^ colony forming units (CFU)/mL (**A**) or host pathogen *P. syringae* pv. *tomato* DC3000 at 8 × 10^4^ CFU/mL (**B**). Samples (rosettes) were collected at 0 dpi and 3 dpi for bacterial quantification. Histograms represent means of four biological replicates. Error bars indicate standard error. Asterisks indicate a significant difference according to Student’s *t* test (* *p*-value < 0.05). All experiments were repeated two times with similar results. (**C**,**D**) Quantification of host and nonhost pathogen multiplication in the *mfdx1-2* mutant. Three-week-old WT and *mfdx1-2* mutant plants were flood inoculated with nonhost pathogen *P. syringae* pv. *tabaci* at 1.6 × 10^4^ colony forming units (CFU)/mL (**C**) or host pathogen *P. syringae* pv. *tomato* DC3000 at 8 × 10^4^ CFU/mL (**D**). Samples (rosettes) were collected at 0 dpi and 3 dpi for bacterial quantification. Histograms represent means of four biological replicates. Error bars indicate standard error. Asterisks indicate a significant difference according to Student’s *t*-test (* *p*-value < 0.05 and ** *p* < 0.01). All experiments were repeated two times with similar results.

To further determine the role of *MFDX1* in plant immunity, we obtained an Arabidopsis homozygous mutant (SALK_033579C) for the *AtMFDX1* gene from the SALK T-DNA collection with a T-DNA insertion located on exon 4, referred here as *mfdx1-1* (Supplementary Materials Figure S2). This *mfdx1* homozygous mutant is a complete knockout (Figure S2). We also obtained another Arabidopsis *mfdx1* mutant (SALK_033569) that showed reduced expression of *AtMFDX1* (Figure S2) and referred as *mfdx1-2*. Both mutants were inoculated along with wild-type Col-0 (WT) with host and nonhost pathogens *P. syringae* pv. *tomato* DC3000 and *P. syringae* pv. *tabaci*, respectively, by flood inoculation method [25,26], samples of which were collected at 0 hpi and 3 dpi for bacterial quantification. In agreement with the data from *NbMDFX1*-silenced *N. benthamiana* plants (Figure S1), both *mfdx1-1* and *mfdx1-2* mutants were significantly more susceptible to a nonhost pathogen at 3 dpi (Figure 2A,C) by supporting around five-fold more bacteria than the WT. Both mutants were also significantly more susceptible to the host pathogen *P. syringae* pv. *tomato* DC3000 (Figure 2B,D).

### 2.3. AtMFDX1 Overexpression Lines Confers Partial Resistance against Host Pathogen P. syringae pv. tomato DC3000

**Figure 3:**
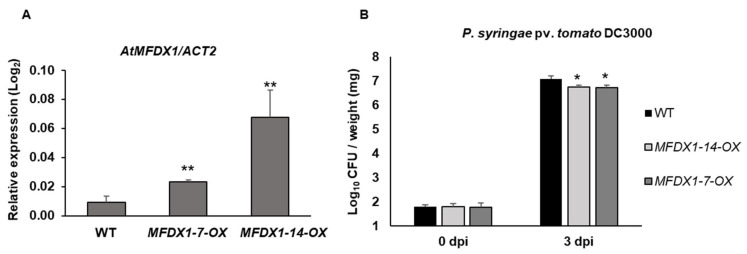
Arabidopsis *AtMFDX1* overexpressor lines are less susceptible to the host pathogen *P. syringae* pv. *tomato* DC3000: (**A**) Relative expression of the *AtMFDX1* gene in the Arabidopsis WT (Col-0) and *AtMFDX1* overexpression lines. Total RNA was isolated from 5-week-old Arabidopsis plants and was subject to RT-qPCR. The expression level was normalized to the *AtACT2* gene. Histograms represent means of four biological replicates. Error bars represent standard error. Asterisks represent statistical significance according to student’s *t* test (** *p*-value < 0.01). All experiments were repeated two times with similar results. (**B**) Quantification of host pathogen multiplication in *AtMFDX1* overexpressor lines. Four-week-old Arabidopsis WT and *AtMFDX1* overexpressor lines (*MFDX1-14-OX* and *MFDX1-7-OX*) were flood inoculated with host pathogen *P. syringae* pv. *tomato* DC3000 at 8 × 10^4^ CFU/mL. Samples (rosettes) were collected at 0 dpi and 3 dpi for bacterial quantification. Histograms represent means of four biological replicates. Error bars indicate standard error. Asterisks indicate significant differences compared to the WT according to Student’s *t* test (* *p*-value < 0.05). hpi, hours post infection; dpi, days post infection.

In order to further confirm the role of *MFDX1* in disease resistance, Arabidopsis lines overexpressing *AtMFDX1* were generated. Expression of *AtMFDX1* was significantly upregulated in two independent overexpression lines, *MFDX1-7*-*OX* and *MFDX1-14-OX* (Figure 3A). Three- to four-week-old *AtMFDX1* (*MFDX1-14-OX* and *MFDX1-7-OX*) overexpression lines and WT were flood inoculated [25] with the host pathogen *P. syringae* pv. *tomato* DC3000. Both overexpression lines were significantly less susceptible to host pathogen infection 3 days after infection in relation to the WT control plants, indicating that *AtMFDX1* may play a positive role in disease resistance (Figure 3B). The observed difference in Log_10_ colony-forming units (CFUs) between the WT and the *MFDX1* overexpression lines is equivalent to a two-fold decrease in bacterial titer for the overexpression lines.

### 2.4. AtMFDX1 Is Ubiquitously Expressed in Leaves and Roots of Arabidopsis

**Figure 4:**
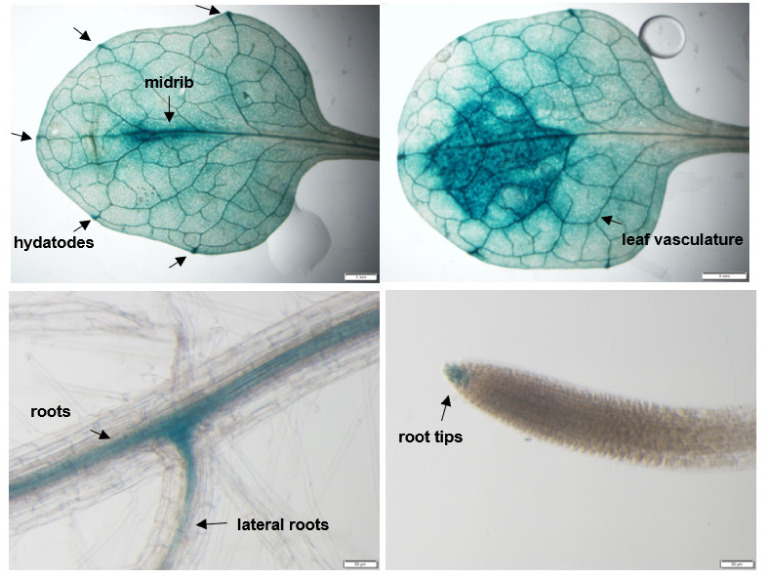
Spatial expression pattern of *AtMFDX1* in Arabidopsis. Histochemical staining of different organs (arrows) of transgenic Arabidopsis seedlings expressing *pMFDX1*:*GUS*. One-month-old *pMFDX1*:*GUS*-expressing Arabidopsis plants were stained with X-Gluc. Photographs of leaf vasculature, roots, lateral roots, root tips and hydatodes were taken 24 h after staining using a stereo microscope. A pronounced accumulation of GUS signal can be observed around the leaf midrib. Scale bars = 50 µm for roots and 1 mm for leaves.

To study the expression pattern of *AtMFDX1* in various Arabidopsis tissues, a 776 bp promoter fragment (from +6 CDS to −770 from ATG site) of *AtMFDX1* was amplified by PCR and cloned into a binary vector as a fusion with a *GUS* gene (*pMFDX1*:*GUS*). The expression pattern of the *GUS* gene under the control of the *AtMFDX1* promoter was determined by GUS histochemical staining of four week-old homozygous stable transgenic Arabidopsis plants expressing *pMFDX1:GUS*. The results showed *GUS* expression in the leaf vasculature, leaf veins, hydathodes, primary and lateral roots, root tips, and around the midrib (Figure 4). These results were consistent with publicly available gene expression data from Genevestigator that shows *AtMFDX1* expression in all plant tissues (Figure S3).

### 2.5. Mfdx1-1 Seedlings Have Reduced Primary Root Size and Secondary Root Number Compared to the WT

**Figure 5:**
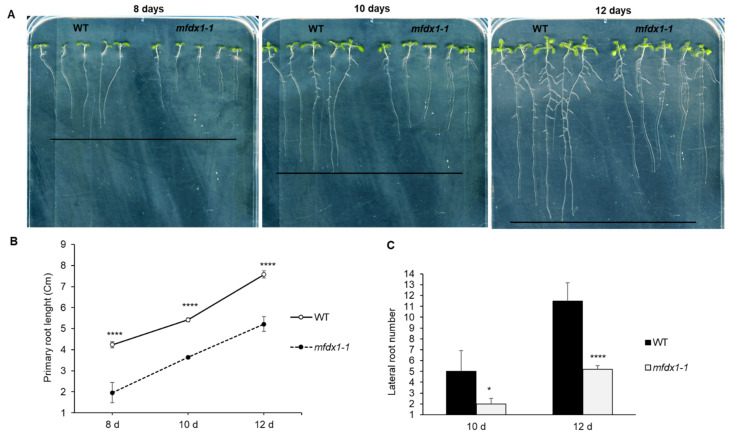
The Fe-S cluster mutant *mfdx1-1* affects primary root length and lateral root number in Arabidopsis. (**A**) Arabidopsis WT (Col-0) and the *mfdx1-1* mutant were germinated and seedlings were grown vertically on ½ MS medium with agar until pictures were taken at 8, 10, and 12 days. Visible differences in root length and lateral root number between the *mfdx1-1* and WT can be observed. Photographs of representative WT and *mfdx1-1* were taken at different days after germination. (**B**) Quantification of primary root length in cm using ImageJ software. Error bars represent SE from 5 seedlings. Asterisks denote statistical significance according to Student’s *t* test (**** *p*-value < 0.0001). Experiment was performed twice with similar results. (**C**) Quantification of lateral root number. Error bars represent standard error from 5 seedlings. Asterisks denote statistical significance according to Student’s *t* test (* *p*-value < 0.05, **** *p*-value < 0.0001). Experiment was performed twice with similar results.

In order to investigate any possible effects of *mfdx1* mutant in Arabidopsis development, we grew the WT and *mfdx1* mutant vertically side-by-side in ½ MS medium plates. A significant difference in primary root length between the *mfdx1-1* mutant and WT could be observed at 8, 10, and 12 days after germination (Figure 5A,B). In addition, we quantified lateral roots and found that the *mfdx1-1* mutant also displayed reduced lateral root number compared to the WT (Figure 5C).

### 2.6. Primary and Secondary Metabolites Differentially Accumulate in mfdx1 Compared to WT upon Pathogen Infection

To further understand the role of *AtMFDX1* in plant immunity, we chose to investigate changes in metabolite accumulation in the *mfdx1-1* mutant compared to WT by GC/MS and LC/MS. We collected the aerial part of Arabidopsis (entire rosette) at 0 hpi (control), 1 dpi, and 3 dpi of *P. syringae* pv. *tomato* DC3000 for metabolite profiling. Using GC/MS to identify primary metabolites (polar fraction), we observed a significant reduction of 5-oxoproline and ornithine (Table 1 and Table S1) in the *mfdx1-1* mutant after pathogen treatment. Ornithine and 5-oxoproline are involved in proline synthesis in mitochondria. Proline and ornithine have been shown to be involved in biotic and abiotic plant stress tolerance [27,28]. A reduction in levels of the defense-related amino acids asparagine and phenylalanine after pathogen infection was also observed in the *mfdx1-1* mutant compared to the WT (Table 1 and Table S1).

To identify secondary metabolites (nonpolar fraction), LC/MS analysis was performed on the same tissues described above for GC/MS. The metabolite profiling results showed that defense related secondary metabolites such as 4-Methylsulfinyl-n-butyl-glucosinolate and 7-Methylsulfinyl-n-heptyl-glucosinolate accumulated significantly less in the *mfdx1-1* mutant compared to the WT, before and after pathogen infection (Table 1 and Table S2).

Overall, the reduction in defense-related metabolites observed in the *mfdx1-1* mutant, such as 5-oxoproline, ornithine, phenylalanine, asparagine, and glucosinolates agrees with the disease-phenotypes reported here for the *mfdx1* mutants and *AtMFDX1* overexpression lines, indicating that the *AtMFDX1* gene is contributing to biotic stress responses by regulating the accumulation of plant defense-related metabolites.

### 2.7. Primary and Secondary Metabolites Differentially Accumulate in AtNFS1 Overexpression Line Compared to WT upon Pathogen Infection

To further understand the mechanism of reduced susceptibility observed in *NFS1.2-18-OX* line [13] against *P. syringae* pv. *tomato* DC3000 at the metabolite level, we investigated changes in metabolite accumulation between a *AtNSF1* overexpressor (*NFS1.2-18-OX*) line and WT control by GC/MS and LC/MS. We collected the aerial part of Arabidopsis (entire rosette) at 0 hpi (control), 1 dpi, and 3 dpi with *P. syringae* pv. *tomato* DC3000. GC/MS analysis of polar fraction metabolites showed the amino acid ornithine significantly accumulated three days after *P. syringae* pv. *tomato* DC3000 infection in the *NFS1.2-18-OX* plants compared to WT control (Table 2 and Table S1). Next, LC/MS analysis indicated the secondary metabolite 8-Methylsulfinyloctyl glucosinolate accumulated significantly in the *NFS1.2-18-OX* line compared to WT control after pathogen infection (Table 2 and Table S2).

### 2.8. Essential Plant Nutrients Differentially Accumulate in AtNFS1 Overexpression and mfdx1-1 Lines Compared to WT upon Pathogen Infection

**Figure 6:**
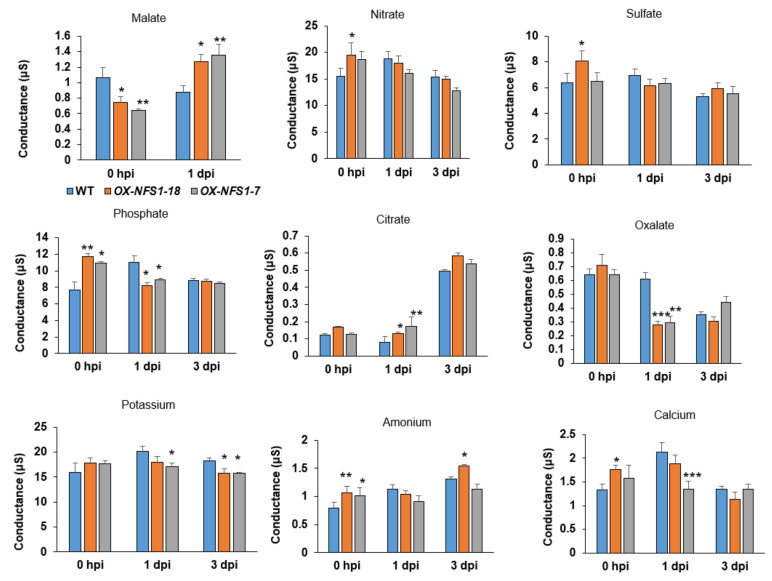
Nutrient accumulation between the *AtNFS1* overexpressor lines and WT plants upon *P. syringae* pv. *tomato* DC3000 infection: The aerial part (leaf and stem) of 5-week-old Arabidopsis seedlings was collected from four plants (*n* = 4) between the WT and *AtNFS1* overexpression lines *NFS1.2-18-OX* and *NFS1.2-7-OX* at different time points (0 hpi, 1 dpi, and 3 dpi) upon flood-inoculation with host pathogen *P. syringae* pv. *tomato* DC3000 (8 × 10^4^ CFU/mL). Metabolites from these samples were extracted and levels of several essential nutrients (cations and anions) were monitored by ion chromatography. Histograms represent means of four biological replicates. Error bars indicate standard error. Asterisks indicate significant differences according to Student’s *t* test (* *p-*value < 0.05, ** *p-*value < 0.01, *** *p-*value < 0.001). hpi, hours post infection; dpi, days post infection.

Plant essential nutrients can help pathogens grow and multiply in the apoplast. We therefore monitored nutrient pool dynamics in the *NFS1.2-18-OX* line, *mfdx1-1* mutant and WT plants at 0 h, 1 day, and 3 days after *P. syringae* pv. *tomato* DC3000 infection by ion chromatography. Several nutrients, such as phosphate, nitrate, sulfate, oxalate, ammonium, calcium, and potassium significantly accumulated in the *NFS1.2-18-OX* line compared to WT at 0 h basal levels (Figure 6). However, 1 day after pathogen infection, the trend was reversed with reduced nutrients in the *NFS1.2-18-OX* line, compared to the WT. Interestingly, for some nutrients like malate and citrate, which are part of the mitochondrial citric acid cycle, the trend was the opposite, with reduced levels at 0 h basal control and increased levels one day after *P. syringae* pv. *tomato* DC3000 infection. Sodium and magnesium did not show any significant change before or after pathogen infection. For the *mfdx1-1* mutant, we did not observe significant changes for most nutrients, but rather a significant decrease in a few nutrients after pathogen infection like magnesium, calcium, potassium and ammonium, and an increase in levels of citrate at 0 h control (Figure S4). Together, these data suggest that *AtNFS1* and *AtMFDX1* could regulate accumulation of metabolites and nutrients upon pathogen infection as part of plant defense response to infection.

## 3. Discussion

Fe-S clusters are protein cofactors involved in catalysis, electron transport, and the sensing of ambient conditions required for several plant physiological processes such as amino acid metabolism, DNA replication and development [10]. We have previously shown that Fe-S cluster genes *AtNFS1* and *AtFH*, when mutated or silenced, exhibited increased susceptibility to host and nonhost pathogens [13]. In this study, we identified and characterized another mitochondrial Fe-S cluster gene, *AtMFDX1*, for its role in host and nonhost disease resistance in plants. We also performed metabolite profiling of a *AtNFS1* overexpression line and *mfdx1-1* mutant in relation to the WT control after host pathogen infection.

The fact that the Fe-S cluster proteins are part of the electron transport chain in energy-generating organelles such as chloroplasts and mitochondria, makes them suitable for developmental and stress response studies [13]. Possible physiological roles of Fe-S cluster components remain largely unknown [11]. The *mfdx1-1* mutant seedlings investigated in this study displayed an altered root developmental phenotype in seedlings compared to the WT plants in Arabidopsis, with shorter primary root length and decreased lateral root number. Overall, this suggests a role for the *MFDX1* gene in regulating growth and development, possibly by impairing mitochondria function, or alternatively, by affecting growth indirectly through the electron transport chain or other mitochondria energy cycles, such as the citric acid cycle [10]. Nevertheless, this hypothesis needs to be tested further.

For the *mfdx1-1* mutant, several primary metabolites involved in biotic and/or abiotic stresses such as phenylalanine, asparagine, 5-oxoproline, and ornithine were significantly reduced when compared to WT upon host pathogen infection. Accumulation of 5-oxoproline and ornithine has been shown to be a common response in abiotic and biotic stress responses [28,29,30]. Proline catabolism occurs in the mitochondria through the action of specialized enzymes like ProDH, PDH1, PDH2, OAT, and P5CDH [31], or from ornithine in the mitochondria [29,32]. The role of *Ornithine delta-aminotransferase* (*OAT*) and *Proline dehydrogenase1* (*ProDH1*) was previously demonstrated in nonhost and basal disease resistance [33].

In Arabidopsis, several abiotic stresses like drought, salinity, cold and also heavy metal exposure can trigger ornithine and proline accumulation [28,31]. It is possible that the mitochondrial protein MFDX1 is involved in abiotic stresses through the ornithine pathway, specifically in the mitochondria by affecting pools of ornithine and 5-oxoproline during proline catabolism.

We also found significant reduction of amino acid asparagine in the *mfdx1-1* mutant compared to the WT even before pathogen accumulation and 3 days after pathogen accumulation. Asparagine levels have been previously correlated with resistance in tomato [34]. Asparagine also accumulates during seed germination and biotic and abiotic stresses [35,36,37]. Similarly, we also observed reduction in phenylalanine levels 3 days after infection in the *mfdx1-1* mutant compared to the WT. The defense phytohormone salicylic acid can be synthesized partly by phenylalanine [38]. Increased levels of phenylalanine have been previously correlated to increased pathogen resistance [39].

In addition to *mfdx1-1* mutant, we also observed accumulation or reduction in the levels of some primary metabolites in the *AtNFS1* overexpression line upon *P. syringae* pv. *tomato* DC3000 infection when compared to WT. Ornithine levels were almost three-fold higher on the third day after infection in the *AtNFS1* overexpression line compared to the WT (Table 1). In addition, proline level was also slightly induced in the *AtNFS1* overexpression line compared to the WT one day after pathogen infection (Table 1). These data suggest that AtNFS1 could potentially affect proline synthesis and catabolism in the mitochondria and that further experiments will be required in the future to show the precise mechanism.

We also found that the secondary metabolite accumulated significantly after pathogen infection in the *AtNFS1* overexpression line when compared to WT control (Table 1). Glucosionolates are sulfur- and nitrogen-containing secondary metabolites derived from different amino acids like alanine, methionine, tryptophan and others that have been shown to be involved in plant defense responses [40,41,42]. Interestingly, we found an opposite trend for the nonhost pathogen susceptible *mfdx1-1* mutant, with reduced levels of 4-Methylsulfinyl-n-butyl-glucosinolate and 7-Methylsulfinyl-n-heptyl-glucosinolate after pathogen infection. Additionally, levels of 3-Hydroxy-4-methoxycinnamic acid were also reduced in the *mfdx1-1* mutant.

Several mechanisms involving nonhost disease resistance have been proposed [23]. It is generally accepted that nonhost resistance has specific pathways affecting disease resistance, but it also shares some mechanisms with host resistance such as hypersensitive response, reactive oxygen species accumulation, and programmed cell death [20]. While host resistance is known for more canonical modes of response like nucleotide-binding site leucine-rich repeat (NBS-LRR) receptor proteins, several non-canonical genes involved in a multitude of cellular processes like specific metabolic pathways or hormone responses can also affect disease resistance, such as *PEN1* [43], *GOX* [44], *PSS1* [45], and *NFS1* [13], among others. *MFDX1* is similar to the latter; it likely affects disease resistance in a non-canonical way by changing pools of disease-related metabolite levels and, possibly, the expression of defense-related genes. Expression of several Fe-S cluster genes (Figure 1) are significantly modulated upon pathogen attack, indicating a transcriptional reprogramming of several components of this pathway during defense responses.

Furthermore, we found several essential nutrients differentially accumulated between the *AtNFS1* overexpression line and the WT control upon pathogen infection. This indicates that AtNFS1 could potentially affect nutrient recycling upon infection to prevent pathogen multiplication in the apoplast. We observed that levels of phosphate, nitrate, sulfate, oxalate, ammonium, calcium, and potassium were higher in the *AtNFS1* overexpression line compared to WT prior to pathogen infection, but that their levels decreased upon infection in the overexpression line. In contrast, we did not observe a significant change for most nutrients between the *mfdx1-1* mutant and the WT. However, we did observe a significant reduction for some nutrients after pathogen treatment like potassium, magnesium, calcium, and ammonium. For TCA cycle derived nutrients such as malate and citrate, the trend was the opposite, with reduced levels at 0 h time point and increased levels one day after *P. syringae* pv. *tomato* DC3000 infection in the *AtNFS1* overexpression line compared to WT control. These results suggest that NSF1 may play a role in diverting nutrient resources in plants upon pathogen infection. While some nutrients may be decreased upon infection as a strategy to starve apoplastic localized pathogens, other nutrients such as malate or citrate may be increased as an energy storage, possibly by an increase in redox potential in the electron transport chain. Another possibility is that changes in levels of nutrient pools upon pathogen infection could be an indirect effect due to a general stress response, instead of a coordinated regulatory response. However, further experiments will be needed to confirm this.

In conclusion, the genetic, transcriptomic, and metabolomic data presented here suggests a new role for mitochondrial Fe-S cluster gene *MFDX1* in plant immunity. Arabidopsis *mfdx1* mutants were more susceptible to host and nonhost pathogens when compared to WT, whereas *MFDX1* overexpression lines were less susceptible to host pathogen when compared to WT. We observed reduction of biotic and/or abiotic stress-response related metabolites such as ornithine, 5-oxoproline, asparagine, phenylalanine, and glucosinolates in the *mfdx1-1* mutant compared to WT plants. In addition, the *mfdx1-1* mutant displayed decreased primary root length compared to the WT. These results suggest a new role for the *AtMFDX1* gene in plant defense responses. In contrast to *mfdx1-1* metabolite data, we observed accumulation of defense-related metabolites in the *AtNFS1* overexpression line. Together, the data presented here reinforces a new role of Fe-S clusters in biotic stress responses in plants.

## 4. Materials and Methods

### 4.1. Plant Material

Arabidopsis plants were grown on ½ MS media in a controlled chamber maintained at 22 °C constant temperature and 50% relative humidity with a 12 h day/night cycle. For experiments involving soil-grown plants, agar germinated plants were transferred to pots containing Metro-Mix 360 two weeks after germination and maintained at same conditions as described above in a growth chamber. Both Arabidopsis *mfdx1-1* mutant line (SALK_033579C) and *mfdx1-2* (SALK_033569) were obtained from the ABRC stock on 13 May 2017 (https://abrc.osu.edu/, accessed on 10 September 2020) [46]. The *mfdx1* mutant seeds were grown in ½ MS media plates containing kanamycin (50 µg/mL) and genotyped using PCR to confirm homozygosity (Table S3).

### 4.2. Cloning and Arabidopsis Transformation

The coding sequence (CDS) of *AtNFS1* and *AtMFDX1* were amplified by PCR from Arabidopsis cDNA (1 µg) with Phusion High-Fidelity DNA Polymerase (New England Biolabs, Ipswich, MA, USA), purified using QIAquick PCR Purification Kit (Qiagen, Valencia, CA, USA) to remove any primer dimers and incubated with Invitrogen BP clonase to generate an entry vector (pDONR207) by transformation of TOP10 (Invitrogen, Carlsbad, CA, USA) competent cells. The product from BP reaction was introduced into the following binary vectors containing the *Cauliflower mosaic virus* promoter (*CaMV 35S*) that drives the gene construct pMDC32 for overexpression lines via LR reaction (LR clonase, Invitrogen) and then transformed into *Agrobacterium tumefaciens* strain GV3101 for stable transformation in Arabidopsis using the floral dip method [47]. All cloned genes were checked by sequencing using Sanger sequencing. For GUS histochemical studies, a 776 bp promoter region of *AtMFDX1* was amplified by PCR from genomic DNA (100 ng) and cloned into the GUS reporter binary vector pMDC162 by GATEWAY cloning and stably-transformed into Arabidopsis in the same way as described above. All primers used in this work can be found in Table S3.

### 4.3. Pathogen Infection Assays

We used the flood inoculation method that mimics natural infection for our disease assays in Arabidopsis [25,26,48]. In brief, four-week-old plants grown in ½ MS media were incubated for 5 min with 40 mL of a bacterial suspension with 0.015% Silwet L-77, containing either one of the following strains: *P. syringae* pv. *tomato* DC3000 (host) or *P. syringae* pv. *tabaci* (nonhost) at the concentrations of 8 × 10^4^ and 1.6 × 10^6^ colony forming units (CFU), respectively. Disease symptoms were observed at five days after infection with *P. syringae* pv. *tabaci* at CFU = 8 × 10^6^. We collected infected tissues at 0 h, 1, and 3 days after the infection and weighed each plant for normalization of CFU. Four plants per time point (*n* = 4) per genotype were used. Whole seedlings were placed in tubes containing grinding beads and 200 µL of sterile water and ground for 1 min to obtain a homogeneous suspension. Suspensions were 10-fold serially diluted for plating. Aliquots of 10 µL were then plated on KB medium with the appropriate selection antibiotic and incubated at 28 °C for two days, after which CFU were counted [49,50]. Statistical analyses were done using Student’s *t* test of the differences between two means of log-transformed data or by one-way ANOVA using the GraphPad Prism software.

### 4.4. Histochemical GUS Staining

GUS histochemical staining of Arabidopsis transgenic lines expressing *pMDX1:GUS* was done using the chromogenic substrate X-gluc according to Hull and Devic (1995) [51]. Whole Arabidopsis plants or specific tissue were submerged in a 2 mM X-gluc solution of 100 mM sodium phosphate, pH 7.0, 10 mM EDTA, 1 mM K_3_Fe(CN)_6_, and 0.1% Triton X-100, applied in a vacuum for 2 min, and then incubated at 37 °C overnight. The following day, the tissues were cleared by successive washes in 70% ethanol to remove chlorophyll for visualization under an Olympus SZX 12—a fluorescent stereo microscope.

### 4.5. RNA-Seq Experiment

Total RNA from Arabidopsis was isolated from 60 mg leaf tissue using Spectrum™ Plant Total RNA Kit (Sigma–Aldrich, St. Louis, MO, USA), and residual DNA was removed from each RNA sample by DNase treatment using TURBO DNA-free Kit (Thermo Fisher Scientific, Waltham, MA, USA) and then purified by RNeasy MinElute Cleanup Kit (Qiagen, Hilden, Germany). The quality and profile of the RNA samples were checked on an Agilent 2100 Bioanalyzer (Agilent Technologies, Santa Clara, CA, USA) using an Agilent RNA 6000 Nano Kit (Agilent Technologies). The RNA samples were quantified on Qubit 2.0 Fluorometer using Qubit RNA BR Assay Kit (Invitrogen, Waltham, MA, USA). RNA-seq libraries were prepared using TruSeq Stranded mRNA Sample Prep Kits (Illumina, San Diego, CA, USA). Briefly, mRNA was purified from 1 µg of total RNA, fragmented and converted to double-stranded DNA for sequencing. Individual libraries were uniquely indexed using TruSeq RNA Single Indexes (Illumina) and pooled in equimolar ratio. The pooled libraries were sequenced on a Hiseq4000 sequencing machine (Illumina).

The sequencing quality of the reads generated were examined by FastQC software (v0.10.1) (http://www.bioinformatics.babraham.ac.uk/projects/fastqc/, accessed on 10 September 2020) on 10 September 2020 and only high-quality samples were used. The filtered clean reads were mapped to the Arabidopsis genome (Araport11, release 2016 downloaded from https://www.araport.org/, accessed on 10 September 2020) to estimate raw counts and effective read lengths by Salmon tool v0.7.2 [52]. All samples displayed a high mapping rate to Arabidopsis genome (>95%). Raw counts from Salmon were normalized across all samples by median normalization, and differential expression was estimated using the DESeq2 module from the Bioconductor R package [53] and an in-house Perl script. Gene expression was quantified as FPKM (fragments per kilobase of exon per million fragments mapped) values, and identified differentially expressed genes at 12 h, 1 day, and 3 days were required to have a False-Discovery Rate (FDR) <0.05 compared to the 0 h time point control.

### 4.6. RNA Extraction and Real-Time Quantitative RT-PCR (RT-qPCR)

Total RNA was isolated from 50 mg leaves using RNeasy Mini Kit (Qiagen), followed by TURBO DNase treatment (Invitrogen). At least 1 µg of RNA was used for cDNA synthesis using SuperScript III reverse transcriptase (Invitrogen) according to the manufacturer’s instructions. Amplicons were amplified from cDNA using gene specific primers, and their relative gene expression values were normalized to the *AtACTIN2 (ACT2)* gene for Arabidopsis samples and *NbACTIN1* gene for *N. benthamiana* using the comparative CT method [54]. RT-qPCR was performed in a Bio-Rad instrument (Eppendorf, Hauppauge, NY, USA) using SYBR Green (Applied Biosystems, Foster City, CA, USA) to monitor dsDNA synthesis. The program used to perform reactions was as follows: 95 °C for 5 min, and extension at 72 °C for 20 s, annealing at 60 °C for 1 min; repeat steps two and three 39 times. To assess amplicon quality, the melting curve was generated at 65 °C until 95 °C with 0.5 °C increments for 5 s.

### 4.7. Metabolite Profiling by GC-MS and LC/MS

Arabidopsis leaf tissue was collected from 5-week-old plants (*n* = 4) for each of the three genotypes used (wild-type [WT], *NFS1.2-18-OX*, and *mfdx1* mutant), at different time points (0, 1, 3 dpi) upon flood-inoculation with *P. syringae* pv. *tomato* DC3000 at 8 × 10^4^ CFU/mL. These samples were lyophilized, ground, and weighed and metabolites were extracted with 80% methanol containing umbelliferone as the internal standard, before being freeze-dried and stored at 80 °C until use. An aliquot of the methanol fraction was removed for secondary metabolites analysis; chloroform and water containing ribitol (polar internal standard) were added separately to the samples. Samples were sonicated and incubated after each addition; separation of the phases was achieved by centrifugation. The polar layer was removed, dried, and re-suspended in 50 µL pyridine with 15 mg/mL methoxyamine-HCl and *N*-Methyl-*N*-(trimethylsilyl)trifluoroacetamide (MSTFA) +1% Trimethylchlorosilane (TMCS) (Pierce Biotechnology, Rockford, IL, USA). After incubation, samples were vacuum centrifuged at 3000 rpm briefly and transferred to a glass insert for analysis. Secondary metabolites were analyzed by ultra-performance liquid chromatography (UPLC) coupled to a Waters Premier hybrid quadropole time-of-flight mass spectrometer, as described by Lei et al. (2019) [55]. Polar metabolites were analyzed using an Agilent 7890B GC system coupled to a high-resolution Agilent 7200A Q-TOF. The data were collected in EI mode. Samples were injected at a 1:30 split ratio. The injector temperature and the MS interface were set at 280 °C. The GC separation was achieved with a temperature program starting at 80 °C for 2 min, then ramped up to 5 °C/min to 315 °C and held for 12 min on a 60 m × 0.25 mm id, 0.25 μM DB-5MS capillary column using He as the carrier gas at 1.2 mL/min. The spectral data were acquired at 5 Hz, and the mass range was 50–750 *m*/*z*. Data were analyzed using Agilent MassHunter and MSDIAL [56] (Ver. 2.8) software. Metabolites were identified through spectral and retention time matching, with authentic compounds using an in-house custom library augmented with a library from Riken.

### 4.8. Ion Profiling by Ion Chromatography (IC)

Part of the leaf samples collected for the metabolite experiment (as described above) were used for ion profiling to measure nutrients. For ion chromatography (IC) measurements, metabolites were extracted from powdered, freeze-dried materials, and samples were processed as described [57] with modifications. Ten mg of each leaf sample was cut into small pieces and ground to fine powder using glass beads. Five mg of ground tissue was mixed with water and incubated in a shaker for 1 h. Samples were sonicated for 20 min and filtered using a 0.2 μM filter attached to a syringe and placed in a plastic vial for analysis. Chromatographic separation was achieved on a Dionex ICS-5000 IC system (Thermo Fisher Scientific) using a Dionex CS12A Ion Pac (2 × 250 mm) analytical column with AG12A guard column (2 × 50 mm) for cations, or a Dionex AS11 Ion Pac (2 × 250 mm) analytical column with AG11 guard column (2 × 50 mm) for anions. Ions were eluted using gradient elution at a flow rate of 0.3 mL/min and detected with a self-regenerating suppressor conductivity detector. Column temperature was maintained at 30 °C and injection volume was 25 μL. The cation eluent source was a Dionex EGC III Methanesulphonic acid eluent generator cartridge (Thermo Fisher Scientific). Elution of the cations was achieved with the following gradient: 12 mM to 20 mM in 7 min, then held at 20 mM from 7 to 15 min. The anion eluent source was a Dionex EGC KOH cartridge (Thermo Fisher Scientific). The elution of anions was achieved with the following concentration gradient: 6mM from 0 to 1 min, ramped from 6mM to 45 mM in 9 min, and then 45 mM to 55 mM in 2 min. Quantification was achieved using Chromeleion 7.2 SR4 Software (Thermo Fisher Scientific).

## Figures and Tables

**Table 1:** Ratio of primary and secondary metabolites accumulation (*mfdx1-1*/WT) upon *P. syringae* pv. *tomato* DC3000 infection.

Metabolite	0 dpi ^1^	1 dpi ^1^	3 dpi ^1^	*p*-Value ^2^		
GC/MS						
5-Oxoproline	0.70	0.38	0.82	0.79	0.04	0.21
Ornithine	1.85	0.85	0.57	0.08	0.65	0.04
Phenylalanine	1.48	1.12	0.54	0.45	0.82	0.00
Asparagine	0.51	1.63	0.28	0.22	0.63	0.00
LC/MS						
4-Methylsulfinyl-n-butyl-glucosinolate	0.86	0.87	0.49	0.12	0.24	0.004
7-Methylsulfinyl-n-heptyl-glucosinolate	0.62	0.46	0.76	0.49	0.005	0.19
3-Hydroxy-4-methoxycinnamic acid	1.34	0.89	0.57	0.20	0.47	0.004

^1^ Values are average between 3–4 biological replicates displayed as ratio between metabolite measurements in the *mfdx1-1* mutant and WT. dpi, days post infection. ^2^ *p*-values represent significant differences according to Student’s *t* test between different measurements.

**Table 2:** Ratio of primary and secondary metabolites accumulation (*NFS1.2-18-OX* / WT) upon *P. syringae* pv. *tomato* DC3000 infection.

Metabolite	0 dpi ^1^	1 dpi ^1^	3 dpi ^1^	*p*-Value ^2^		
GC/MS						
5-Oxoproline	0.94	0.18	1.01	0.92	0.01	0.91
Ornithine	1.89	0.95	2.93	0.10	0.87	0.03
Proline	0.40	1.38	0.68	0.01	0.40	0.52
LC/MS						
8-Methylsulfinyl-n-octyl glucosinolate	0.92	2.31	5.30	0.19	0.13	0.03

^1^ Values are average between 3–4 biological replicates displayed as ratio between metabolite measurements in the *AtNFS1* overexpression line and WT. dpi, days post infection. ^2^ *p*-values represent significant differences according to Student’s *t* test between different measurements.

## Data Availability

All data generated by this study is available upon request.

## Supplementary Materials

The following are available online at https://www.mdpi.com/article/10.3390/ijms22137147/s1, Figure S1: *NbMFDX1*-silenced plants are more susceptible to infection by the nonhost pathogen *P. syringae* pv. *tomato* T1 than the control., Figure S2: The *mfdx1* and *mfdx-2* mutants have completely abolished or reduced *MFDX1* expression, respectively compared to the WT. Figure S3: Expression of *AtMFDX1* in different organs in Arabidopsis. Figure S4: Comparison of nutrient accumulation between the *mfdx1-1* mutant and WT plants upon *P. syringae* pv. *tomato* DC3000 infection. Table S1: GC/MS primary metabolite data in the *NFS1.2-18-OX* line, mfdx1-1 mutant and the WT at 0 h, 1 d and 3 days upon host pathogen infection., Table S2: LC/MS data secondary metabolites in the *NFS1.2-18-OX* line, mfdx1-1 mutant and the WT at 0h, 1d and 3 days after host pathogen infection., Table S3: All primers used in this work.
