# Supplementary material for: The Arabidopsis Iron-Sulfur (Fe-S) Cluster Gene MFDX1 Plays a Role in Host and Nonhost Disease Resistance by Accumulation of Defense-Related Metabolites
Source: Int J Mol Sci. 2021 Jul 1;22(13):7147. doi: 10.3390/ijms22137147 (PMC8269267; doi:10.3390/ijms22137147)
Supplement: Supplementary file 1 [file ijms-22-07147-s001.zip › Supplemental Figures.pptx]

## Slide 1
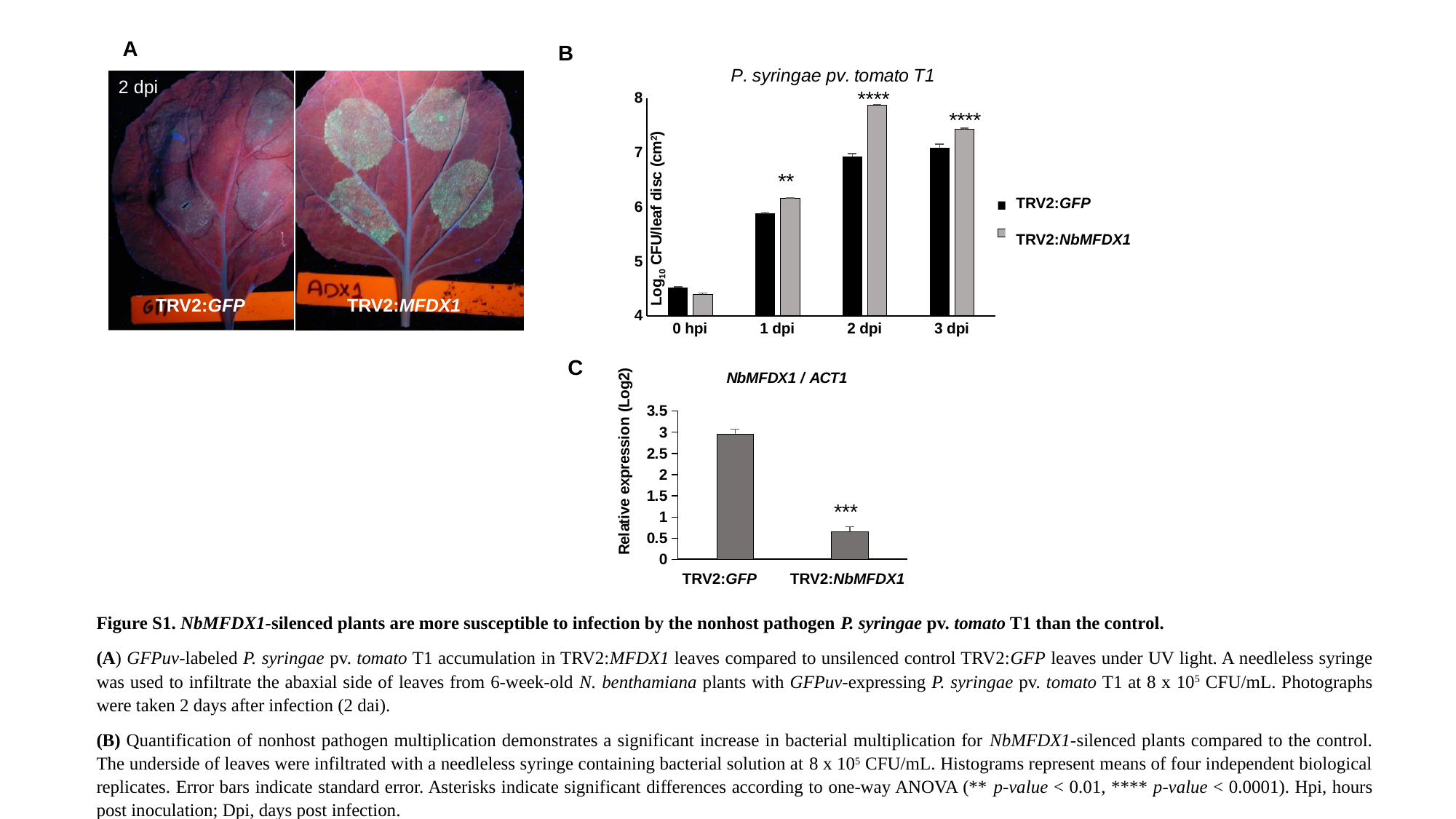

A
B
### Chart: P. syringae pv. tomato T1
| Category | TRV2:GFP | TRV2:MFDX1 |
|---|---|---|
| 0 hpi | 4.510445237607918 | 4.389738119021592 |
| 1 dpi | 5.885098606978875 | 6.159109951652493 |
| 2 dpi | 6.928205764210959 | 7.87289594875849 |
| 3 dpi | 7.083774238030261 | 7.429012755452438 |2 dpi
TRV2:GFP
TRV2:NbMFDX1
TRV2:GFP
TRV2:MFDX1
C
### Chart: NbMFDX1 / ACT1
| Category | |
|---|---|
| TRV2:GFP | 2.957730296356069 |
| TRV2:NbMFDX1 | 0.6561672742503547 |TRV2:GFP TRV2:NbMFDX1
Figure S1. NbMFDX1-silenced plants are more susceptible to infection by the nonhost pathogen P. syringae pv. tomato T1 than the control.
(A) GFPuv-labeled P. syringae pv. tomato T1 accumulation in TRV2:MFDX1 leaves compared to unsilenced control TRV2:GFP leaves under UV light. A needleless syringe was used to infiltrate the abaxial side of leaves from 6-week-old N. benthamiana plants with GFPuv-expressing P. syringae pv. tomato T1 at 8 x 10^5^ CFU/mL. Photographs were taken 2 days after infection (2 dai).
(B) Quantification of nonhost pathogen multiplication demonstrates a significant increase in bacterial multiplication for NbMFDX1-silenced plants compared to the control. The underside of leaves were infiltrated with a needleless syringe containing bacterial solution at 8 x 10^5^ CFU/mL. Histograms represent means of four independent biological replicates. Error bars indicate standard error. Asterisks indicate significant differences according to one-way ANOVA (** p-value < 0.01, **** p-value < 0.0001). Hpi, hours post inoculation; Dpi, days post infection.
(C) Relative gene expression of the NbMFDX1 gene between TRV2:NbMFDX1 and TRV2:GFP (control) inoculated plants. Three weeks after inoculating N. benthamiana plants with TRV2 clones, RNA was isolated from leaves and subject to RT-qPCR. The expression level was normalized to NbACTIN1 expression. Histograms represent means of three biological replicates. Error bars indicate standard error. Asterisk indicates significant differences according to student’s t test (***p-value < 0.001). All experiments were repeated two times with similar results.

## Slide 2
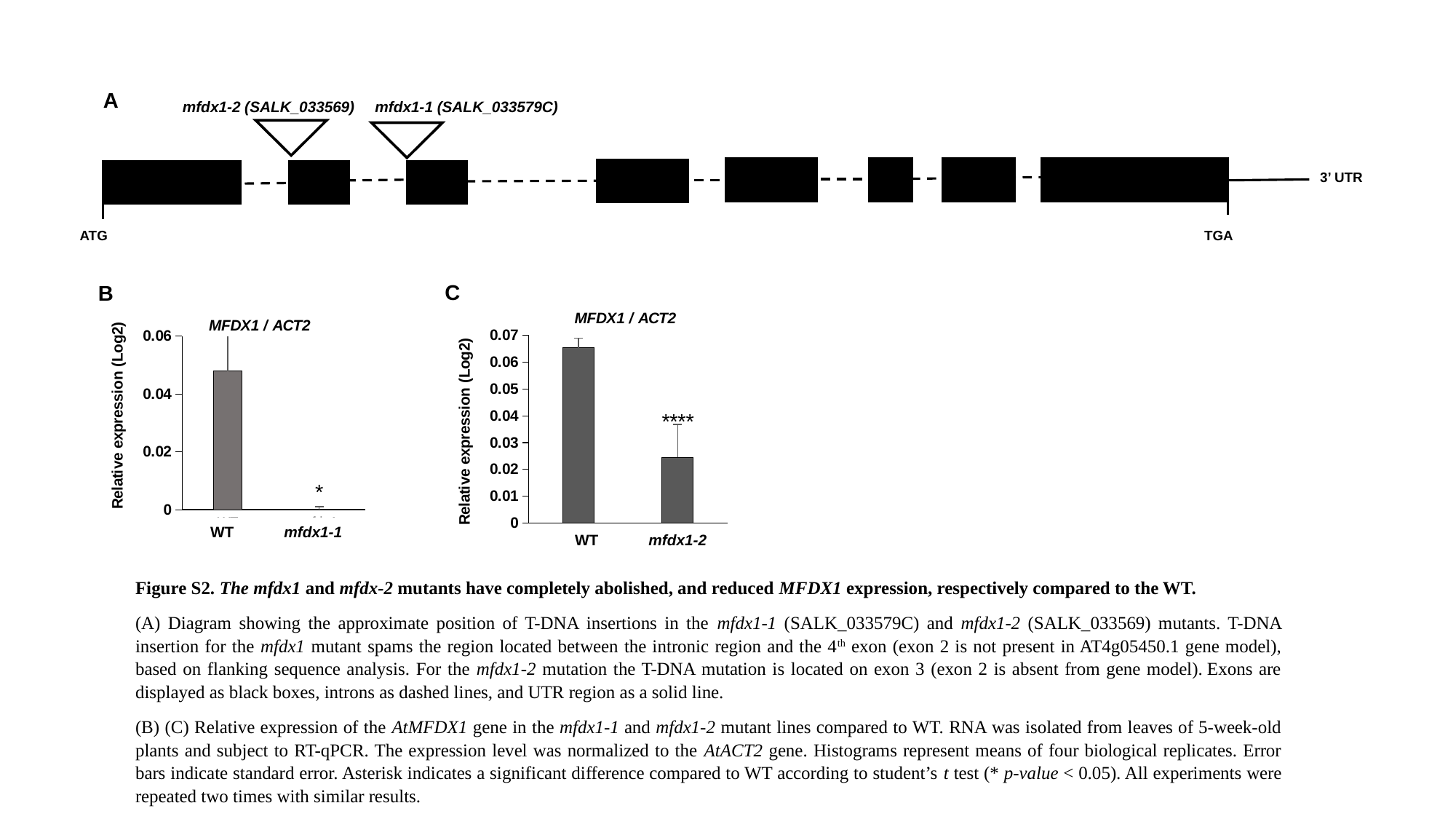

A
mfdx1-2 (SALK_033569)
mfdx1-1 (SALK_033579C)
3’ UTR
TGA
ATG
C
B
### Chart: MFDX1 / ACT2
| Category | |
|---|---|
| WT | 0.06534822674020575 |
| mfdx1-1 | 0.024518822890507403 |
### Chart: MFDX1 / ACT2
| Category | |
|---|---|
| WT | 0.04791761676906432 |
| mfdx1 | 0.0 |WT mfdx1-1
WT mfdx1-2
Figure S2. The mfdx1 and mfdx-2 mutants have completely abolished, and reduced MFDX1 expression, respectively compared to the WT.
(A) Diagram showing the approximate position of T-DNA insertions in the mfdx1-1 (SALK_033579C) and mfdx1-2 (SALK_033569) mutants. T-DNA insertion for the mfdx1 mutant spams the region located between the intronic region and the 4th exon (exon 2 is not present in AT4g05450.1 gene model), based on flanking sequence analysis. For the mfdx1-2 mutation the T-DNA mutation is located on exon 3 (exon 2 is absent from gene model). Exons are displayed as black boxes, introns as dashed lines, and UTR region as a solid line.
(B) (C) Relative expression of the AtMFDX1 gene in the mfdx1-1 and mfdx1-2 mutant lines compared to WT. RNA was isolated from leaves of 5-week-old plants and subject to RT-qPCR. The expression level was normalized to the AtACT2 gene. Histograms represent means of four biological replicates. Error bars indicate standard error. Asterisk indicates a significant difference compared to WT according to student’s t test (* p-value < 0.05). All experiments were repeated two times with similar results.

## Slide 3
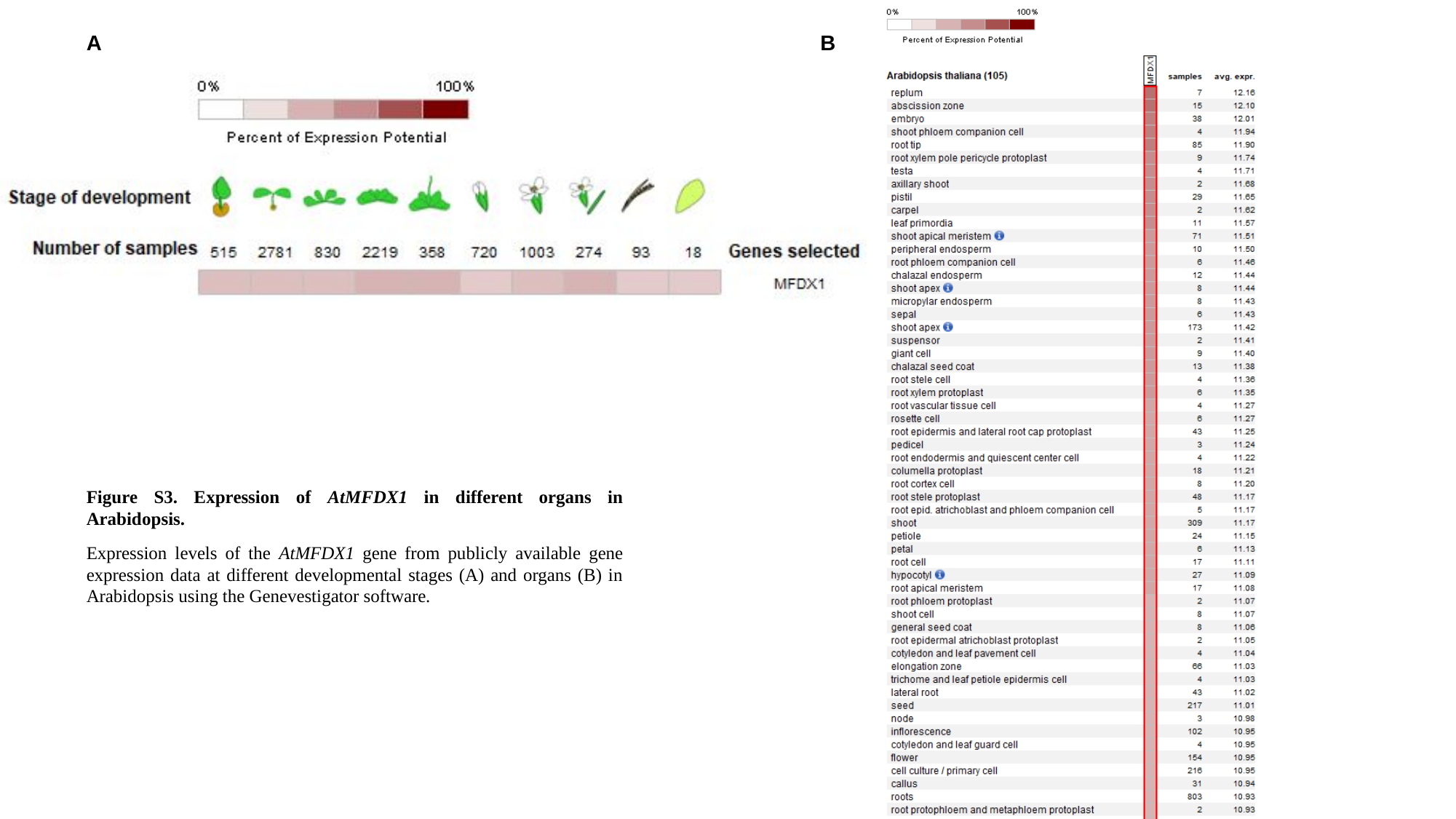

A
B
Figure S3. Expression of AtMFDX1 in different organs in Arabidopsis.
Expression levels of the AtMFDX1 gene from publicly available gene expression data at different developmental stages (A) and organs (B) in Arabidopsis using the Genevestigator software.

## Slide 4
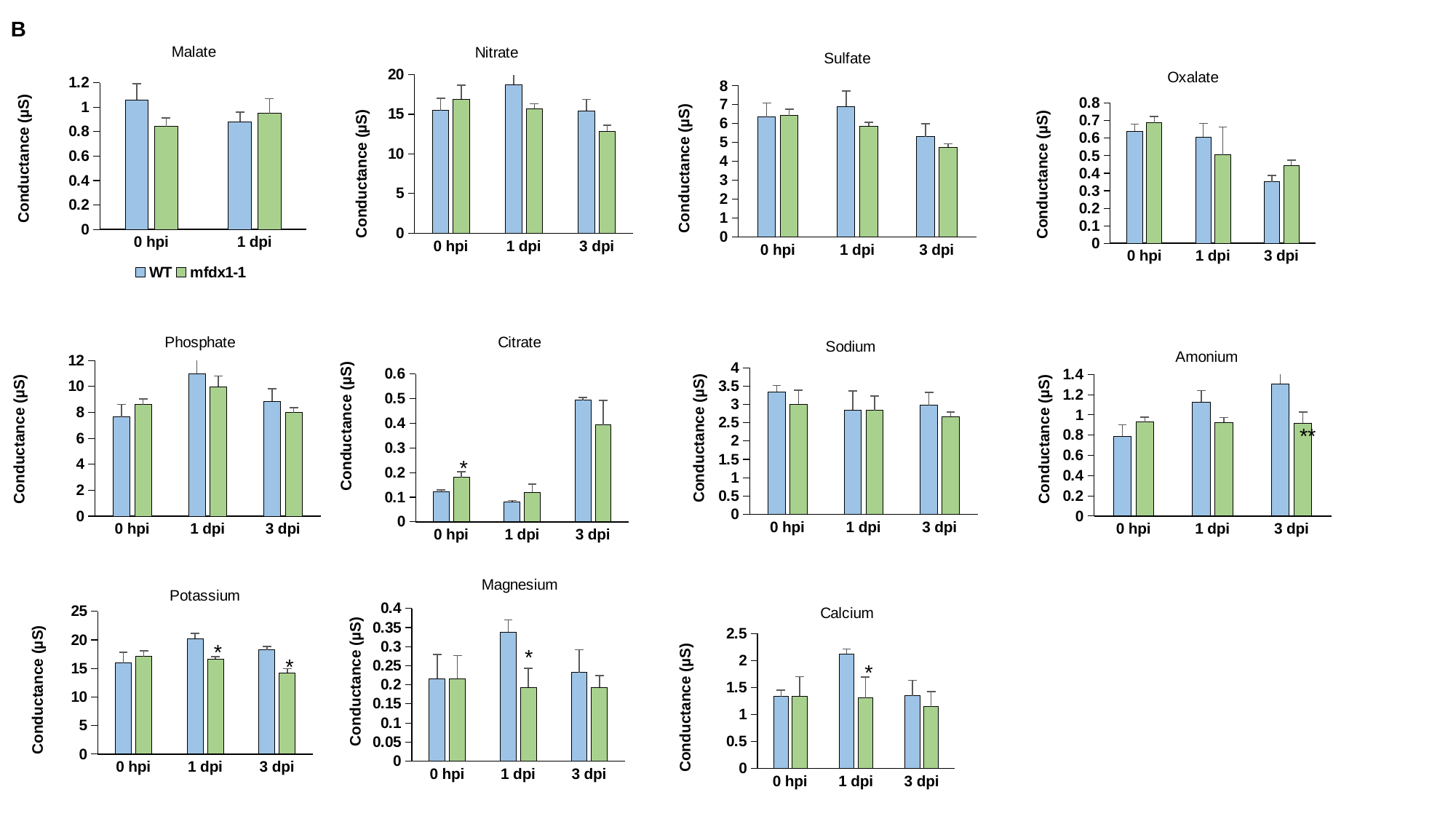

B
### Chart: Nitrate
| Category | WT | mfdx1 |
|---|---|---|
| 0 hpi | 15.485975 | 16.897966666666665 |
| 1 dpi | 18.75055 | 15.723233333333333 |
| 3 dpi | 15.4091 | 12.8407 |
### Chart: Malate
| Category | WT | mfdx1-1 |
|---|---|---|
| 0 hpi | 1.0602 | 0.8420333333333333 |
| 1 dpi | 0.881075 | 0.949733333333333 |
### Chart: Sulfate
| Category | WT | mfdx1 |
|---|---|---|
| 0 hpi | 6.344074999999999 | 6.428733333333334 |
| 1 dpi | 6.907575 | 5.8551 |
| 3 dpi | 5.302066666666667 | 4.742999999999999 |
### Chart: Oxalate
| Category | WT | mfdx1 |
|---|---|---|
| 0 hpi | 0.6406 | 0.6872333333333334 |
| 1 dpi | 0.6067 | 0.5040000000000001 |
| 3 dpi | 0.35203333333333336 | 0.44493333333333335 |Conductance (µS)
Conductance (µS)
Conductance (µS)
Conductance (µS)
### Chart: Phosphate
| Category | WT | mfdx1 |
|---|---|---|
| 0 hpi | 7.649733333333334 | 8.614666666666666 |
| 1 dpi | 10.993400000000001 | 9.980533333333334 |
| 3 dpi | 8.8475 | 7.976700000000001 |
### Chart: Citrate
| Category | WT | mfdx1 |
|---|---|---|
| 0 hpi | 0.12273333333333332 | 0.17989999999999998 |
| 1 dpi | 0.081 | 0.12033333333333333 |
| 3 dpi | 0.4941 | 0.39336666666666664 |
### Chart: Sodium
| Category | WT | mfdx1 |
|---|---|---|
| 0 hpi | 3.3410499999999996 | 3.0079000000000007 |
| 1 dpi | 2.8498750000000004 | 2.8342333333333336 |
| 3 dpi | 2.972566666666667 | 2.6631 |
### Chart: Amonium
| Category | WT | mfdx1 |
|---|---|---|
| 0 hpi | 0.789975 | 0.9284 |
| 1 dpi | 1.128175 | 0.9258000000000001 |
| 3 dpi | 1.3054000000000001 | 0.9193666666666666 |Conductance (µS)
Conductance (µS)
Conductance (µS)
Conductance (µS)
### Chart: Magnesium
| Category | WT | mfdx1 |
|---|---|---|
| 0 hpi | 0.216225 | 0.21566666666666667 |
| 1 dpi | 0.338425 | 0.19333333333333333 |
| 3 dpi | 0.23199999999999998 | 0.1933666666666667 |
### Chart: Potassium
| Category | WT | mfdx1 |
|---|---|---|
| 0 hpi | 15.96165 | 17.123233333333335 |
| 1 dpi | 20.2047 | 16.64033333333333 |
| 3 dpi | 18.27325 | 14.249466666666669 |
### Chart: Calcium
| Category | WT | mfdx1 |
|---|---|---|
| 0 hpi | 1.33195 | 1.3428333333333333 |
| 1 dpi | 2.1247249999999998 | 1.3097666666666667 |
| 3 dpi | 1.3508 | 1.1475333333333333 |Conductance (µS)
Conductance (µS)
Conductance (µS)

## Slide 5
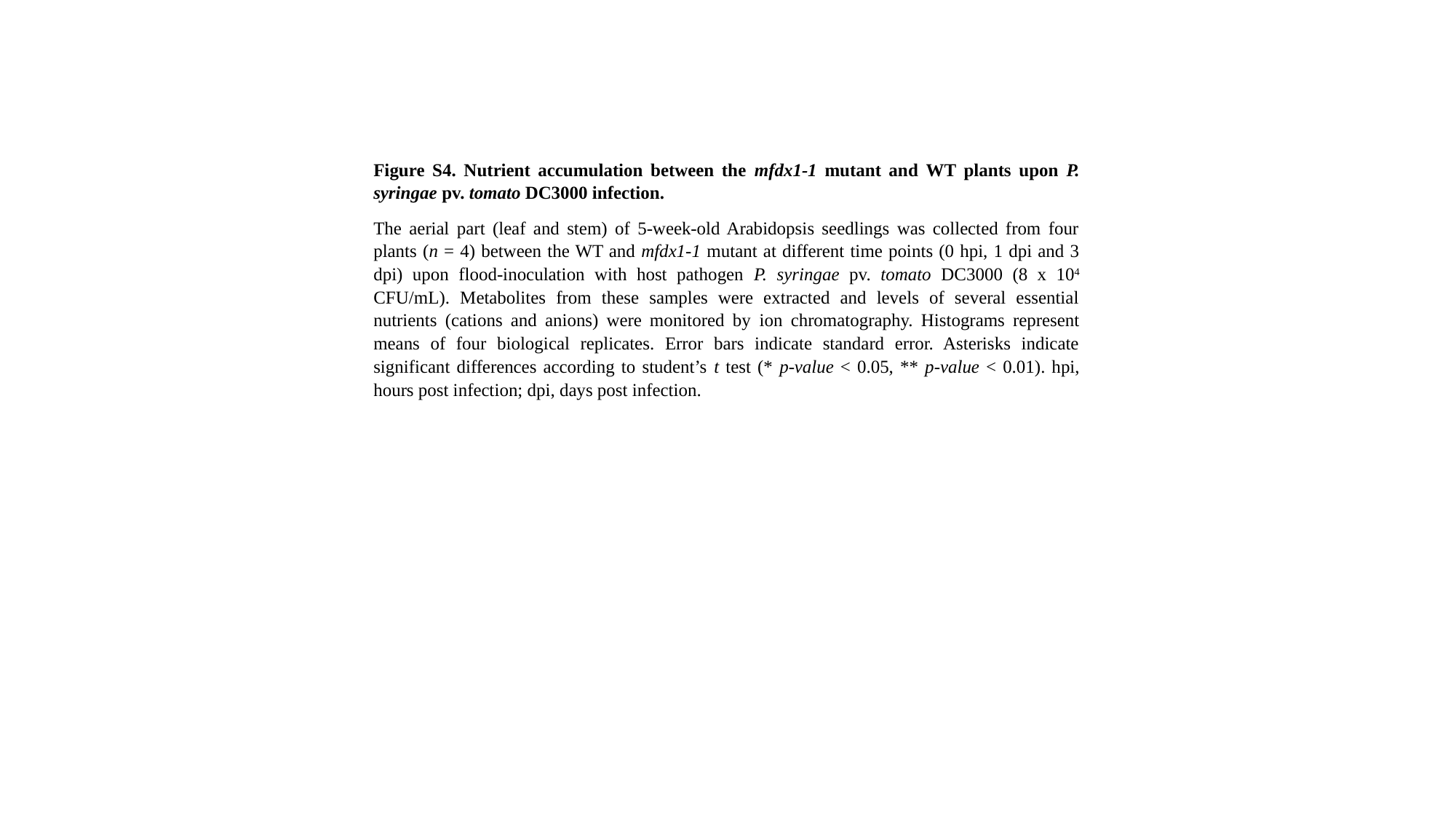

Figure S4. Nutrient accumulation between the mfdx1-1 mutant and WT plants upon P. syringae pv. tomato DC3000 infection.
The aerial part (leaf and stem) of 5-week-old Arabidopsis seedlings was collected from four plants (n = 4) between the WT and mfdx1-1 mutant at different time points (0 hpi, 1 dpi and 3 dpi) upon flood-inoculation with host pathogen P. syringae pv. tomato DC3000 (8 x 10^4^ CFU/mL). Metabolites from these samples were extracted and levels of several essential nutrients (cations and anions) were monitored by ion chromatography. Histograms represent means of four biological replicates. Error bars indicate standard error. Asterisks indicate significant differences according to student’s t test (* p-value < 0.05, ** p-value < 0.01). hpi, hours post infection; dpi, days post infection.
